# Some Practical Points about Porcelain

**Published:** 1907-06-15

**Authors:** E. H. Shannon


### Some Practical Points about Porcelain.

BY E. H. SHANNON, D. D. S.

To overcome the difficulties of securing a good matrix from cervical cavities on the labial surfaces, cut the platinum in a curved form at the top and bend it to about the form of the labial surface of the tooth; with wet cotton gently packed into the cavity, the matrix will be carried to every part of the cavity, and if the cervical part of the matrix has been pushed up under the gum margin, there will be sufficient matrix to take the cervical wall accurately. The burnishers will complete the adaptation.

I make burnishers to suit special cases by fusing gold onto the points of old excavators or pluggers that have been previously fluxed. The gold knobs can be shaped to any form desired. I like some made like inverted cone burs for flat or angular surfaces.

Use surgeons' artery forceps, ground to suitable points for holding the matrix; they hold securely and are handy to manipulate.

Use powdered silex to rest the matrix on while fusing. Don't re-burnish the matrix after it is partially filled with porcelain, and don't take to a glaze the first body. To get an accurate impression of a root stump, take Dentalac and roll into a stick the size of a lead pencil, and draw the end down to a point. Heat the small end until soft enough, and press it firmly onto the root stump and hold until it hardens. Make a counter model by forcing this impression into softened mouldine, into which metal may be poured to secure the root stump. Coat this die with whiting dissolved in alcohol, and pour over it a counter die. Into this a platinum plate can be swaged that will fit the root stump.

Use the furnace to modify the form or color of stock teeth to give them an artistic appearance, especially where some natural teeth remain.

A broken tooth may be mended or restored if only the new porcelain be added and the baking properly done.

